# Effects of the Application of Information Technology to E-Book Learning on Learning Motivation and Effectiveness

**DOI:** 10.3389/fpsyg.2021.752303

**Published:** 2021-09-13

**Authors:** Li Sun, Cheng En Pan

**Affiliations:** Business & Tourism Institute, Hangzhou Vocational and Technical College, Hangzhou, China

**Keywords:** information technology, ebook teaching, learning motivation, learning effectiveness, extrinsic orientation

## Abstract

Along with the rapid development of electronic technology, the appeal of information technology to students and teachers in domestic and international information education has become universal. Education-related departments aim to positively cultivate the professional information knowledge and skills of teachers. The use of information technology in instruction allows students to enhance their creativity and learning motivation. A total of 232 college students from Fujian Province participated in the experimental research. The results show the following: (1) the application of information technology to e-book teaching could enhance a sense of achievement of students in self-directed learning, and students could answer test questions in a confidential and relaxed manner; (2) the application of information technology to e-book teaching activates teaching flexibility, and many teaching models, such as teaching by wandering around, interactive teaching, and blended teaching, are therefore derived to enhance the richness of teaching; (3) the application of information technology to e-book teaching bridges the distance between instructors and students and leads to a deeper understanding of learning conditions of students, expanding the possibilities for content planning and teaching models. The results give rise to suggestions enhancing enjoyment in learning and promoting higher motivation and more effective teaching.

## Introduction

The rapid evolution of mobile information technology and multimedia technology results in diversified teaching models for information education. The advance of mobile information technology changes traditional fixed-point teaching to a mobile learning situation. Mobile learning is the learning method allowing learners to acquire information or knowledge through mobile devices and the internet without being restricted to time and space. It allows learners to get rid of the limits of time, space, hardware, and platform, and the characteristics of rapid and easy access to global information have the integration between multimedia materials and tools present better interactivity and mobility to further provide an open and timely system with learner-centered and learner-controlled learning situation (Lin et al., 2017). Instructors teaching or inspecting students can enhance immediate interactions and improve the concentration of students. Multimedia technology drives e-book integrated learning, and e-books can include multimedia audio and visual effects, thereby enhancing information education. The rapid development of computer technology means that “books” are presented in new ways. A simple key command calls up an “e-book” with the desired information, containing not only text and symbols but also offering the possibility of beautiful music and rich and complex images and animation effects. Textbooks of students have become e-books, and future textbooks will be published electronically.

Domestic and international information education follows the rapid development of electronic technology. There is a consensus among students and teachers on the appeal of information technology. Education-related departments have begun cultivating professional information knowledge of teachers, enabling teachers to work remotely with students. The widespread application of technology means that modern students are accustomed to electronic information and audiovisual stimulation and are ready to adapt their learning styles and needs to the digital age. The change in learning styles from static to dynamic means that teaching models now involve information equipment. E-books, in particular, are attractive because they offer the advantage of multimedia audio and visual effects. However, research on reading motivation, exploring the enhancement of motivation by using information technology in the form of e-book learning, has been rare. Accordingly, the present research examines the effect of the application of information technology to e-book learning on learning motivation and effectiveness. It is expected that the use of information technology in e-book learning could enable students to access information and that creativity and learning motivation will be enhanced.

## Literature Review and Hypotheses

Leong et al. (2019) described e-books as the combination of images, sounds, storage media, an interface, and editing software. When used as part of a planned teaching design, e-books enlarge the learning window due to the interaction of senses of the learners. Such interactions can help learners to understand and can establish the concepts of the knowledge to be taught. The effect is more efficient than traditional teaching media and can better enhance the motivation of learners and interests. Ali (2018) compared e-books with paper books and indicated that e-books outperformed paper books on presentation patterns, number of users, sensory learning, material attributes, evaluation feedback, costs, and searchability. Therefore, the application of e-book integrated learning with lively and diverse materials (images and sound effects) could enhance the learning motivation and learning effectiveness of students. Khamis (2019) regards e-books as an interactive medium that is unlike paper as a medium. The text, sounds, images, and video elements present specific content in a dynamic, multimedia manner that breaks away from traditional paper or audible books. Accordingly, the following hypothesis is proposed.

**H1**: Using information technology for e-book learning would affect learning motivation.

Saini and Kaur (2019) pointed out that the interaction in e-books is not simply the interaction between students and e-books. It can include two-way communication between teachers and students. In interactive learning, teachers and students can communicate opinions bilaterally. Teachers can easily give timely guidance, making students more likely to pay attention than in general teaching and enhancing the effectiveness of learning. This is a big advantage of interactive learning. Baharuddin and Hashin (2020) stated that e-books could offer students diverse learning situations, which, in comparison with general teaching, could better enhance self-directed learning and reading motivation of students, provide interaction experience between students and media, enhance logical thinking and integration ability of students, and allow students to learn with their senses (sight, hearing, and touch). The authors found that e-books enhance learning comprehension, the construction of learning strategies, and the promotion of learning achievement. Phadung and Dueramae (2018) showed that e-books, including animation, allow students to specify abstract concepts. E-books offer clear and easily accessible explanations of events and phenomena, allowing students to understand the background to events and enhancing their investigative abilities. The teaching content becomes rich and diversified, allowing for closer interaction between teachers and students, the enhancement of student participation in the learning process, the achievement of targeted learning through repeated practice, and the promotion of learning effectiveness. Consequently, the following hypothesis is proposed.

**H2**: Using information technology for e-book learning would affect learning effectiveness.

Yee and Zainuddin (2018) identified a positive effect on the learning motivation and learning effectiveness of students. Elenein (2019) proposed that enhancing the learning motivation of students could significantly promote the learning effectiveness of students. Interested students would understand and participate in learning activities with a positive learning attitude. Teaching plans should take the learning motivation of students into account and optimize factors in the learning motivation of students to enhance learning motivation and effectiveness. Rachels and Rockinson-Szapkiw (2018) considered that students with high learning motivation present more clearly defined goals and a strong desire to learn the content well. They have higher expectations for results and better self-effectiveness. Sritharan (2018) found better performance among students with high learning motivation and better performance among students with intrinsic motivation than among those with extrinsic motivation. As a result, the following hypotheses are proposed.

**H3**: Learning motivation reveals significant and positive effects on learning effect in learning effectiveness.
**H4**: Learning motivation shows remarkable and positive effects on learning gains in learning effectiveness.

## Methodology

### Measurement of Research Variables

#### Learning Motivation

Referring to Huang and Chang (2019), learning motivation is divided into intrinsic orientation and extrinsic orientation.

#### Learning Effectiveness

Referring to Chang et al. (2019), learning effect and learning gains are utilized in this study.

### Research Subject and Sampling Data

A university in Fujian Province was selected as the research object. A total of 232 students participated in the 16 weeks (3 h per week for a total of 48 h) experimental research on the application of information technology to e-book learning. SPSS was used to analyze the data, and factor analysis and reliability analysis, regression analysis, and ANOVA were applied to test the hypotheses.

### Analysis Method

Analysis of variance was applied to identify the difference in using information technology for e-book learning in learning motivation and learning effectiveness. Regression analysis was applied to identify the relationship between learning motivation and learning effectiveness.

## Results

### Effects of the Application of Information Technology to E-Book Learning on Learning Motivation and Learning Effectiveness

(1) Difference analysis of using information technology for e-book learning in learning motivation

Analysis of variance was used to discuss, analyze, and explain the difference in learning motivation between the application of information technology to e-book learning and traditional teaching methods using paper books. The results in Table 1 show that information technology for e-book learning (4.13) appears to have a higher intrinsic orientation than traditional teaching with paper books (3.75), and information technology for e-book learning (4.34) reveals higher extrinsic orientation than traditional teaching with paper books (3.69). Therefore, H1 is supported.

**Table 1:** Difference analysis of using information technology for e-book learning in learning motivation.

**Variable**		**F**	***P***	**Scheffe *post hoc***
Using information technology for e-book learning	Intrinsic orientation	29.634	0.000^**^	E-book (4.13) > paper books (3.75)
	Extrinsic orientation	25.377	0.000^**^	E-book (4.34) > paper books (3.69)

** *p < 0.01*.

(2) Difference analysis of using information technology for e-book learning in learning effectiveness

Analysis of variance was applied to discuss the difference in using information technology for e-book learning in learning effectiveness, i.e., analysis and explanation of the application of information technology to e-book learning and traditional teaching with paper books. Table 2 shows a higher learning effect of the application of information technology to e-book learning (4.07) than traditional teaching with paper books (3.51) and higher learning gains of using information technology for e-book learning (4.26) than traditional teaching with paper books (3.84). Therefore, H2 is supported.

**Table 2:** Difference analysis of using information technology for e-book learning in learning effectiveness.

**Variable**		***F***	***P***	**Scheffe *post hoc***
Information technology for e-book learning	Learning effect	23.962	0.000^**^	E-book (4.07) >paper books (3.51)
	Learning gains	31.451	0.000^**^	E-book (4.26) >paper books (3.32)

** *p < 0.01*.

### Correlation Analysis of Learning Motivation and Learning Effectiveness

#### Correlation Analysis of Learning Motivation and Learning Effect

The analysis results in Table 3 reveal notable effects of intrinsic orientation (β = 0.215, *P* < 0.001) and extrinsic orientation (β = 0.247, *P* < 0.001) on learning effect. Therefore, H3 is supported.

**Table 3:** Analysis of learning motivation and learning effectiveness.

**Dependent variable→**	**Learning effectiveness**			
**Independent variable↓**	**Learning effect**		**Learning gains**	
**Learning motivation**	**β**	***P***	**β**	***P***
Intrinsic orientation	0.215	0.000^***^	0.239	0.000^***^
Extrinsic orientation	0.247	0.000^***^	0.224	0.000^***^
*F*	18.631	27.384		
Significance	0.000^***^	0.000^***^		
*R* ^2^	0.269	0.304		
Adjusted *R*^2^	0.242	0.274		

*** *p < 0.001*.

*Data source: self-organized in this study*.

#### Correlation Analysis of Learning Motivation and Learning Gains

Table 3 shows significant effects of intrinsic orientation (β = 0.239, *P* < 0.001) and extrinsic orientation (β = 0.224, *P* < 0.001) on learning gains. Therefore, H4 is supported.

## Discussion

In the application of information technology to e-book learning, the one-to-one computer screen could more easily induce learning interests and attention of students. The specific animation and audio allow students to clearly understand theoretical concepts. The presentation of e-books could better enhance the thinking and critical ability of students. Furthermore, the diversified extension activities, compared to traditional homework assignments, are more acceptable for students, thereby enhancing the integration of theoretical concepts (therefore, teachers could increase the unit and time with the integration of ebooks to specify the parts which are not easily presented with textbooks. It could develop the effectiveness of ebooks, and the timely integration of multimedia information technology into teaching shows positive effects on learning motivation and learning effectiveness of students). Teachers could make use of suitable e-books and could integrate multimedia information technology into their teaching. This would bring about positive effects on learning motivation and the learning effectiveness of students. Teaching with materials closely related to the life of students could make the information more acceptable to students. Teachers, in the teaching process, should familiarize themselves with the e-book content and the related knowledge. They should control the time spent on working with e-books and should interact and discuss with students in a timely manner. They should avoid long e-book reading sessions that might result in learning pressure on students (In the interactive discussion between teachers and students, it is necessary to give sufficient time for students to discuss and think about teaching contents; the next part of ebook teaching should be played after full understanding of students. In the interactive discussion, teachers should timely guide changing virtual situations in ebooks into real knowledge situations for students, or, with the multimedia production ability, present the illustration of important knowledge in ebooks with special effects, or assist with authentic pictures or texts to have students clarify correct scientific knowledge and prevent students overly personifying scientific knowledge). In addition, teachers, in the interaction process, should encourage students at appropriate times to present personal ideas, and they should encourage students to engage in critical thinking and questioning about e-books.

## Conclusion

The research findings show that students in the experimental group, starting from pieces of ideas on theoretical concepts and moving to more complete ideas, could eventually apply their skills to theoretical concepts in a flexible manner. Theoretical concepts of the students are shown to be enhanced. Therefore, teaching using the application of information technology to e-book learning is specific and effective and could enhance the theoretical concepts of students. As a result, it is practicable and effective for teachers to apply information technology to e-book learning and to use lively animation with audio and music to attract the attention of students. Not only could this process enhance the learning attitudes of students and the nature of their learning, but it could also have positive effects on learning achievement. In the comparison between the experimental group and the control group, the former appears to have a significantly higher average score on learning motivation, learning essence, and learning achievement than the control group. This research shows that the application of information technology to e-book learning causes it to outperform traditional teaching with paper books in terms of effective teaching.

## Data Availability Statement

The original contributions presented in the study are included in the article/supplementary material, further inquiries can be directed to the corresponding author.

## Ethics Statement

The present study was approved and conducted in accordance with the recommendations of the ethics committee of the Hangzhou Vocational and Technical College, China, with written informed consent being obtained from all the participants. All the participants were asked to read and approve the ethical consent form before participating in the present study. The participants were also asked to follow the research guidelines as set out in the consent form.

## Author Contributions

All authors revised and approved the submitted version of the manuscript. LS performed the initial analyses and wrote the manuscript. CP assisted in the data collection and data analysis. All authors contributed to the article and approved the submitted version.

## Conflict of Interest

The authors declare that the research was conducted in the absence of any commercial or financial relationships that could be construed as a potential conflict of interest.

## Publisher's Note

All claims expressed in this article are solely those of the authors and do not necessarily represent those of their affiliated organizations, or those of the publisher, the editors and the reviewers. Any product that may be evaluated in this article, or claim that may be made by its manufacturer, is not guaranteed or endorsed by the publisher.
